# Trends in the prevalence of grandparents living with grandchild(ren) in selected European countries and the United States

**DOI:** 10.1007/s10433-018-0474-3

**Published:** 2018-05-23

**Authors:** Karen Glaser, Rachel Stuchbury, Debora Price, Giorgio Di Gessa, Eloi Ribe, Anthea Tinker

**Affiliations:** 1 Institute of Gerontology, Department of Global Health & Social Medicine, School of Global Affairs, Faculty of Social Science and Public Policy, King’s College London, London, UK; 2 CeLSIUS, Department of Epidemiology and Public Health, University College London, 1-19 Torrington Place, London, UK; 3 Manchester Institute for Collaborative Research on Ageing, University of Manchester, Manchester, UK

**Keywords:** Multigenerational household, Family support, Older people, Grandparents, IPUMS, ONS Longitudinal Study

## Abstract

Research from the United States has shown significant increases in the prevalence of three-generation households and in households consisting solely of grandparents and grandchildren. Such shifts in household composition, which are associated with socio-economic disadvantage, may reflect the activation of grandparents as a latent network of support in response to social and demographic changes such as rising partnership disruption. However, to date, little is known in Europe about trends in grandparent households or whether these households are also likely to be disadvantaged. Moreover, we know little about how the familistic and defamilised policy environments in Europe may affect the activation of such latent kin networks. Employing the Integrated Public Use Microdata Series—International and the Office for National Statistics’ Longitudinal Study for England and Wales, we used multivariate techniques to investigate changes in prevalence over time in co-residence with a grandchild across Austria, England and Wales, France, Greece, Portugal, Romania, and the United States. We expected increases in grandparent households in Portugal and Greece, familistic societies with few public alternatives to family support. However, only Romania (like the US) showed an increase in the percentage of people aged 40 and over co-residing with their grandchildren in three-generation households between the late 1970s and 2002. Given rises in poverty and limited support for low-income families in Romania, rises in grandparent coresidence may reflect a coping strategy among poorer families to increasing financial hardship. Regardless of the trends, grandparent households in all the countries studied remained associated with socio-economic disadvantage.

**Electronic supplementary material:**

The online version of this article (10.1007/s10433-018-0474-3) contains supplementary material, which is available to authorised users.

## Introduction

It is well established that grandparents play an important role in family life, providing financial, emotional and practical care, and support to their children and grandchildren (Baydar and Brooks-Gunn 1998; Bordone et al. 2017; Di Gessa et al. 2016; Dunifon et al. 2014; Fuller-Thomson and Minkler 2001; Fuller-Thomson et al. 1997; Hagestad 2006; Hank and Buber 2009; Herlofson and Hagestad 2012; Igel and Szydlik 2011; Jendrek 1993; Koslowski 2009; Minkler and Fuller-Thomson 2005; Price et al. Forthcoming). However, increasing survival, higher rates of divorce and relationship breakdown, and public sector retrenchment mean that the role grandparents play in family life is likely to have increased (Dunifon et al. 2014; Hagestad 2006; Herlofson and Hagestad 2012; Murphy 2011). A growing body of work has investigated the involvement of grandparents in grandchild care from a predominantly European comparative perspective (Bordone et al. 2017; Di Gessa et al. 2016; Hank and Buber 2009; Igel and Szydlik 2011; Koslowski 2009; Price et al. Forthcoming). In contrast, there is little comparative research on grandparental coresidence with most work based on single countries, and little analysis of trends for Europe (Albuquerque 2011; Casper and Bryson 1998; Dunifon et al. 2014; Goodman and Silverstein 2002; Minkler 1999; Nandy and Selwyn 2013; Pebley and Rudkin 1999; Pew Research Center 2010; Pew Research Center 2013; Pew Research Center 2014; Prokos and Keene 2012; Selwyn and Nandy 2014). An understanding of variations over time in grandparent coresidence is critical as it is strongly associated with both grandparental child and kinship care (Baydar and Brooks-Gunn 1998; Vandell et al. 2003), and may be either beneficial or detrimental for the health and wellbeing of each generation (Chambers et al. 2017; Dunifon and Kowaleski-Jones 2007; Hayslip and Kaminski 2005; Kreidl and Hubatkova 2014; Mutchler and Baker 2009; Pittman and Boswell 2008; Szinovacz et al. 1999; Tanskanen 2013).

To date, evidence on grandparental coresidence comes largely from the United States (US), which has experienced significant increases in the prevalence of multigenerational and grandparent households since the 1970s (Casper and Bryson 1998; Dunifon et al. 2014; Pew Research Center 2010; Pew Research Center 2013; Pew Research Center 2014), and where grandparent households are associated with socio-economic disadvantage (Dunifon et al. 2014). Data are routinely collected in the US on whether grandparents have ‘primary responsibility’ for raising a grandchild allowing the identification of so-called ‘custodial households’ (Casper and Bryson 1998; Fuller-Thomson et al. 1997; Mutchler and Baker 2004; Pew Research Center 2013). To our knowledge, no national survey in Europe collects these data save for such ‘kinship care’ as might be inferred from coresidence (Nandy and Selwyn 2013; Selwyn and Nandy 2014). European data can, however, distinguish between ‘three-generation households’ (comprising grandparents and grandchildren, with at least one of their parents) and ‘skipped-generation households’ (consisting of grandparents and grandchildren but without the parents) where kinship care can be implied (Casper and Bryson 1998; Mutchler and Baker 2004; Nandy and Selwyn 2013; Pew Research Center 2013; Selwyn and Nandy 2014).

Thus, our research used the Integrated Public Use Microdata Series (IPUMS)—International and the Office for National Statistics’ Longitudinal Study (ONS LS) for England and Wales across three or four time points from the late 1970s onward to investigate trends in the prevalence of people aged 40 or over living with their grandchild(ren) in the selected European countries (Austria, England and Wales, France, Greece, Portugal and Romania) and the United States. Our study also examined the socio-economic and demographic characteristics associated with such individuals and their households and associated changes over time.

## Background

### Trends in grandparent households

Intergenerational coresidence in Western industrialised countries declined dramatically over the course of the 20th century (Pew Research Center 2010; Pew Research Center 2014; Ruggles 2007; Tomassini et al. 2004). However, research from the US shows a significant increase since the 1970s (Pew Research Center 2010; Pew Research Center 2013; Pew Research Center 2014; Wiemers 2014), with a rise in households including at least two adult generations from 12% in 1980 to 18% in 2012, and increases also observed among three-generation and skipped-generation households (Casper and Bryson 1998; Hayslip and Kaminski 2005; Minkler 1999; Pebley and Rudkin 1999; Pew Research Center 2013). In the US, the percentage of children living in a household headed by one or more grandparents rose from 3% in 1970 to 7% by 2008–2010 (with a more precipitous rise after the start of the recession in 2007), suggesting an important increase in the share of grandparents raising or helping to raise grandchildren (Casper and Bryson 1998; Dunifon et al. 2014; Hayslip and Kaminski 2005; Minkler 1999; Murphey et al. 2012; Pebley and Rudkin 1999; Pew Research Center 2013).

In Europe, intergenerational households are less common in Northern than in Southern and Eastern Europe; however, we know little about trends in these household types (Albuquerque 2011; Iacovou and Skew 2011; Koslowski 2009; Tomassini et al. 2004). A Portuguese study showed an increase in the percentage of households with grandparents from 1994 to 2001 from around 7–11% (Albuquerque 2011). In the UK, it is estimated that in 2001, 1% of children co-resided with grandparents and that this proportion doubled between 1991 and 2001 (Nandy et al. 2011). We do not know whether these trends are apparent in other European countries.

### Grandparent households and socio-economic disadvantage

In the US, grandparents living in households with their grandchildren are more likely to be socio-economically disadvantaged compared to other grandparents (Dunifon et al. 2014; Fuller-Thomson and Minkler 2001; Fuller-Thomson et al. 1997; Luo et al. 2012; Minkler and Fuller-Thomson 2005; Mutchler and Baker 2004). This is particularly the case for custodial households where grandparents are disproportionately younger, female, unmarried, African American, not in paid work, with lower educational levels, having more grandchildren, and living in poverty (Dunifon et al. 2014; Fuller-Thomson and Minkler 2001; Fuller-Thomson et al. 1997; Kreider and Ellis 2011; Prokos and Keene 2012). Grandparents in skipped-generation households in particular are more likely to fall below the poverty line (Casper and Bryson 1998; Minkler and Fuller-Thomson 2005; Mutchler and Baker 2004). For example, 26% of households with children under age 18 with at least one grandparent but no parent present were below the poverty level in 2009 in comparison to 16% of households consisting of grandparents and at least one parent (a similar poverty level to that experienced by all households with a child under age 18; Kreider and Ellis 2011). Cultural factors such as race and/or ethnicity, immigration status, and religion are also important; for example, mothers who are Black or Hispanic are more likely to live in three-generation households in comparison to non-Hispanic whites, and married mothers whose parents were immigrants are more likely to also live in such households (Dunifon et al. 2014; Luo et al. 2012; Pilkauskas 2012). However, even in the US, we know little about changes in the relationship between individual demographic and socio-economic factors associated with grandparental coresidence over time.

In Europe, there is limited evidence on the characteristics of grandparent households and on changes over time in these households (Albuquerque 2011; Koslowski 2009; Nandy and Selwyn 2013; Selwyn and Nandy 2014). In the UK, close to half of grandmother kinship caregivers reported a limiting long-term illness (Nandy and Selwyn 2013). In Portugal, as in the US, co-resident grandparent households are more likely to consist of grandmothers rather than grandfathers and are over represented in the lowest income quartile (Albuquerque 2011). In addition, such households also became poorer over time when compared to other households (Albuquerque 2011). A greater understanding of the trends and socio-economic characteristics associated with three-generation or skipped-generation grandparent households is critical for demonstrating any associated social inequalities and improving related policy.

### Explanations for rising grandparent households

Explanations for any rise in grandparent households come, necessarily, largely from the US. Reasons for the increase there (and for the rise in skipped-generation households in particular) have primarily focused on two factors. The first refers to generational needs, especially the support needs of the parent generation due to socio-economic trends (e.g. increases in substance use particularly during the crack cocaine epidemic of the 1980s/1990s, parental incarceration, and financial difficulties) and changes in family life (e.g. rises in single-parenthood and divorce). The second relates to social welfare reforms, such as policy changes aimed at moving mothers from welfare to work, requirements that unmarried teenage mothers live with an adult (i.e. usually a parent), and child welfare reforms including shifts toward kinship rather than public foster care (Baker et al. 2008; Cherlin and Seltzer 2014; Cuddeback 2004; Dunifon et al. 2014; Goodman et al. 2004; Gordon 1999; Hayslip and Kaminski 2005; Minkler 1999; Nandy and Selwyn 2013; Pew Research Center 2013; Smith and Beltran 2001).

Two theories may help to explain rising coresidence with grandparents in the US (Baker et al. 2008): the activation of a latent matrix of kin and structural lag (that is the failure of societal arrangements to respond to rapid social transformations in individual lives) (Baker et al. 2008). The first approach is the idea that socio-demographic change has created a latent matrix of kin support: increases in survival have led to longer years of shared lives across generations resulting in a network of kin that can be called on when needed (Riley and Riley 1993). Under this perspective, multigenerational households may be an adaptive strategy to a wide range of changing family or social circumstances (e.g. parental substance misuse, imprisonment or financial difficulties) (Cherlin and Seltzer 2014). Being fluid and flexible, grandparent households are thought to be particularly well suited to respond to such changes. The second related approach refers to structural lag: structural changes are not able to keep pace with changes to individual or family lives leading to asynchrony between the two (Riley 1994). This may mean that family members with limited resources of time, energy, or materials come under pressure to support the most vulnerable (often children).

### The European policy context

Europe presents a unique setting for such a study given similar socio-economic trends to those seen in the US; for example, increasing prevalence of drug and alcohol misuse, financial crises such as the recent economic recession in 2008–2009, and similar changes to child welfare policies in countries such as the UK. However, these trends are occurring in different policy environments to that of the US. Earlier work shows that the welfare state has an important impact on grandchild care (Bordone et al. 2017; Di Gessa et al. 2016; Igel and Szydlik 2011; Price et al. Forthcoming). We use Saraceno and Keck’s (2010) typology of familialism in welfare state regimes, where policies are indicative of the degree to which individuals are (in)dependent of families with greater dependence on the state or the market (Saraceno and Keck 2010). While it is recognised that this classification scheme is based on specific transfers to children (and to older people) such as childcare arrangements and parental leave, nevertheless the scheme is likely to reflect broader societal values regarding family obligations and responsibilities (Herlofson and Hagestad 2012; Saraceno 2016). Thus, we hypothesise that in societies classified as *familialism by default* (where there are few publicly provided alternatives to family care or financial support in the case of families in need such as in Greece and Portugal) or as *supported familialism* (where families are supported by financial transfers such as in Romania) kin activation in the form of grandparental coresidence will have increased by comparison to societies characterised by *defamilialisation* (where there is greater availability of publicly or market provided services such as in France and to a lesser extent Austria and the UK) (Herlofson and Hagestad 2012; Saraceno 2016; Saraceno and Keck 2010). Moreover, we expect to see greater socio-economic disadvantage associated with grandparent households in countries characterised by *familialism by default*, as families will be more likely to rely on their own resources in times of need given the absence of public support (Saraceno 2016).

## Data and methods

### Census microdata

For Austria, France, Greece, Portugal, Romania, and the US, IPUMS—International (for details see https://international.ipums.org) provided (as far as possible) harmonised and comparable samples of census data; the sampling fraction was 5% for the US, Portugal and France, 10% for Austria, Greece and, Romania, and 33% for France in 2011 using its rolling census for 2009–2013. Where appropriate (that is, for the US data and for France in 2013) weights have been applied to the data to take account of different sampling fractions. All the censuses use the ‘de jure’ rule, enumerating persons usually resident on census day irrespective of their actual location. All the countries chosen had a series of census datasets available from IPUMS, taken around the years 1981 (1977 for Romania), 1991 and 2001, as well as another around 2011 for France, Portugal, and the US.

All these census microdata sets provide representative samples both of private households or dwellings (depending on the country) and of persons. For our analyses, persons living in group quarters were excluded. No census data allow identification of a grandparent unless she/he is coresiding with a grandchild. However, all those used offered the relationship (from a restricted range) of each member to the head of household. We could, therefore, identify a co-resident grandparent in cases where she/he was head of a household and a grandchild (aged 0–17 years) was present, or where she/he was the parent of the household head who in turn had a child (aged 0–17 years) in the household. The proportion of co-resident grandparents thus identified is, therefore, likely to be a small underestimate, since a grandparent could co-reside with a dependent grandchild in a household where, for example, it was the grandparent’s sibling that was the head.

For England and Wales, the ONS LS offers linked census and life events data for a 1% sample of the population of England and Wales from 1971. Census data were used from 1981, 1991, 2001, and 2011. In our analyses, we used households headed by an LS sample member. Co-resident grandparents were identified as for the other datasets, and LS members in communal establishments were excluded from analysis.

### Measures and methods

We analysed the living arrangements of people aged 40 and over, as fewer than 1% of adults under 40 are grandparents (Leopold and Skopek 2015). Our definition of grandchildren excluded those aged 18 years or more (Nandy and Selwyn 2013). Two types of grandparent households were examined: three-generation and skipped-generation.

### Analytical approach

Two analytic strategies were employed: first, descriptive and bivariate analyses examined change over time in the likelihood of people aged 40 and over coresiding with their grandchild(ren) and their individual and household characteristics in each country using t-tests and Chi-square tests as appropriate for the outcome. Unless indicated otherwise, only differences that are statistically significant at the 1% level receive comment. Second, we used multinomial logistic regression given that our outcome has three categories (i.e. not in a grandparent household, three-generation, and skipped-generation household). We report the relative risk ratio that is the risk associated with the selected characteristics of being in a three- or skipped-generation household relative to not being in a grandparent household. We included demographic characteristics such as sex, age, and marital status in order to consider the effects of compositional change in the population over time and to examine changes in their associations with grandparent coresidence. Following earlier studies, we used age and age-squared in the models as we expected the relationship between age and coresidence to become stronger at older ages. This being census data, our socio-economic covariates were limited to educational attainment, employment status (that is employed, not in the labour force including retired individuals and those classified as looking after the home, and unemployed), and whether the individual was foreign-born (Albuquerque 2011; Fuller-Thomson and Minkler 2001; Fuller-Thomson et al. 1997; Kreider and Ellis 2011; Prokos and Keene 2012). We also considered whether the dwelling was owned or rented, identified as an important determinant of coresidence in Central and Eastern Europe (Stephens et al. 2015).

## Results

### Descriptives

**Fig. 1:**
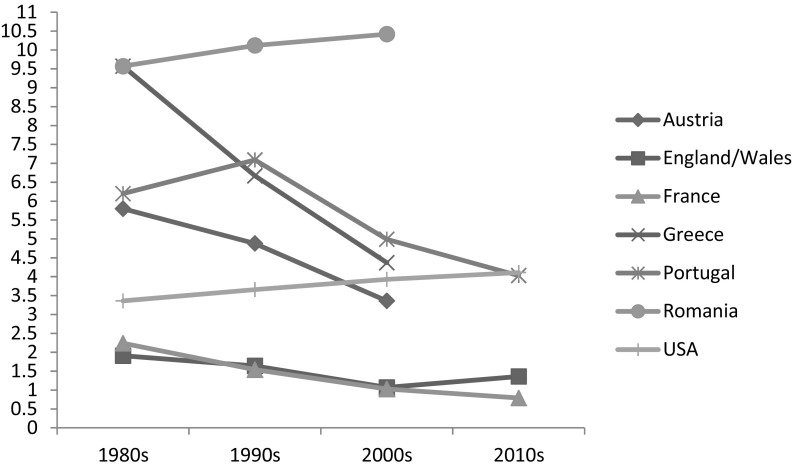
Percentage of people aged 40 years or more coresiding with both adult children and grandchildren (aged 0–17). Source: IPUMS-International (Minnesota Population Center 2017) and ONS LS

**Fig. 2:**
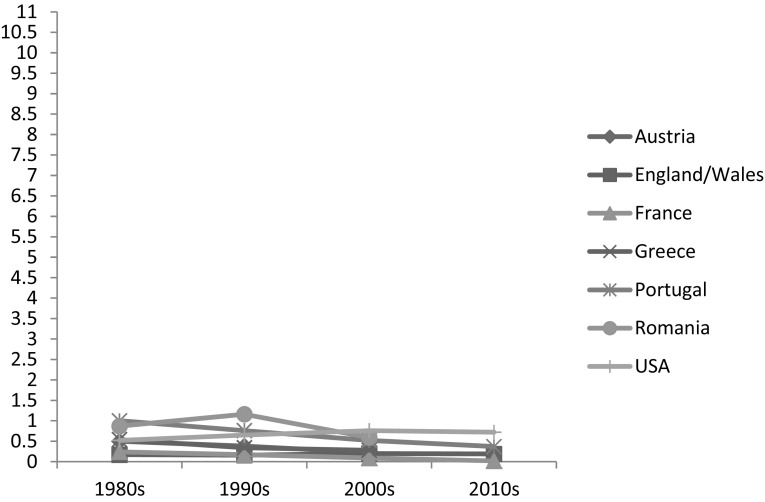
Percentage of people aged 40 years or more coresiding with grandchildren aged 0–17 but with no adult children in the household. Source: IPUMS-International (Minnesota Population Center 2017) and ONS LS

Figure 1 shows that between around 1981 and 2011, in Austria, France, Greece, and Portugal the percentage of people aged 40 or over living in three-generation households with their grandchild(ren) declined. In the US, as expected, this percentage increased somewhat to 4% in 2010; in Romania, it remained relatively high and stable at around 10%. Figure 2 shows trends in skipped-generation households: increases over the period were observed only in England and Wales and the US, though caution is required in interpretation given the small percentages of such households in the countries studied.

Table 1 shows selected characteristics of people aged 40 and over and the percentage who were co-resident grandparents in three-generation households. Across all six European countries considered, the decline observed for the total population is also generally apparent by each of the selected characteristics, with the few exceptions described below.

**Table 1:** Percentage of people aged 40 and over living with child(ren) and grandchild(ren) aged 0–17 by selected adult characteristics, weighted data (Source: IPUMS-International (Minnesota Population Center 2017) and ONS LS)

	Austria			England and Wales				France				Greece			Portugal				Romania			USA			
	1981	1991	2001	1981	1991	2001	2011	1982	1990	1999	2011	1981	1991	2001	1981	1991	2001	2011	1977	1992	2002	1980	1990	2000	2010
	%	%	%	%	%	%	%	%	%	%	%	%	%	%	%	%	%	%	%	%	%	%	%	%	%
Sex																									
Male	4.9	4.3	2.9	1.6	1.4	0.9	1.1	1.7	1.2	0.8	0.6	7.0	5.0	3.4	5.6	5.8	4.1	3.3	7.5	8.6	8.9	2.6	2.9	3.0	3.2
Female	6.5	5.4	3.7	2.2	1.9	1.2	1.6	2.7	1.8	1.2	0.9	12.0	8.2	5.3	6.7	8.2	5.8	4.6	11.4	11.4	11.7	4.0	4.3	4.7	5.0
Age																									
40s	2.3	2.1	1.3	1.1	1.2	0.7	0.8	0.6	0.6	0.4	0.3	1.2	0.8	0.7	2.7	2.0	1.8	1.4	3.7	4.7	4.0	2.8	2.8	2.9	2.7
50s	5.4	5.4	3.2	1.9	2.1	1.2	1.9	1.8	1.5	1.1	0.9	6.1	4.5	3.1	7.1	7.0	4.7	4.5	10.4	12.0	12.9	3.8	5.1	4.8	4.8
60s	7.5	6.7	5.1	2.2	2.0	1.5	1.6	2.9	2.0	1.5	1.2	14.9	9.5	6.8	8.4	10.1	7.4	5.7	14.8	13.3	16.6	3.6	4.4	5.4	5.5
70s	9.0	6.3	5.0	2.6	1.6	1.1	1.6	4.1	2.4	1.4	1.1	22.2	14.1	8.0	8.1	10.9	7.3	5.5	15.3	12.5	11.1	3.4	2.9	4.0	4.7
80s	8.9	5.7	4.0	2.8	1.3	0.7	0.8	4.6	2.4	1.4	0.8	24.8	14.5	7.0	7.6	10.8	6.7	4.5	14.2	10.4	7.2	3.3	2.1	2.7	2.9
90 plus	8.5	5.1	3.4	2.1	0.9	1.5	0.3	4.0	2.3	1.4	0.8	20.4	11.0	4.8	6.0	7.0	5.5	3.2	12.0	7.1	5.1	3.4	1.6	2.1	1.6
Marital status																									
Never-married	1.7	1.3	1.1	0.3	0.4	0.8	0.5	0.5	0.5	0.4	0.4	0.5	0.0	0.3	1.9	0.7	2.0	1.8	3.5	2.9	4.7	1.0	1.6	2.1	2.2
Married	5.1	4.8	3.2	1.4	1.5	1.0	1.4	1.5	1.2	0.9	0.7	7.2	5.1	3.6	5.7	5.5	4.0	3.7	7.5	9.2	9.6	2.6	3.2	3.5	3.8
Divorced/sep.	2.7	2.6	2.7	2.5	1.6	1.0	1.3	1.9	1.4	1.0	0.8	6.0	3.6	5.6	5.9	7.2	5.0	3.2	7.0	6.0	6.4	5.1	4.8	4.8	4.8
Widowed	11.1	8.1	6.2	4.8	3.1	1.7	2.0	6.1	3.7	2.4	1.7	27.5	18.4	10.9	11.5	17.9	12.1	8.3	20.2	16.2	16.3	6.6	5.7	6.6	6.9
Education																									
Less than primary	na	na	na	na	na	na	na	3.2	2.6	2.0	2.0	15.9	12.7	9.5	6.6	8.0	6.2	5.8	11.6	14.1	16.5	8.0	10.5	14.9	14.5
Primary	8.0	7.3	5.5	2.1	1.9	1.3	1.6	1.8	1.4	1.0	0.8	6.9	5.7	4.6	3.1	3.0	2.6	2.4	7.6	9.8	12.4	4.4	5.6	6.8	8.0
Secondary	2.7	2.8	2.1	0.8	0.8	0.8	1.6	0.7	0.7	0.7	0.5	2.0	1.4	1.5	2.4	2.3	1.7	1.4	3.7	4.9	5.7	2.4	3.2	3.7	4.0
University	1.1	1.1	0.9	0.4	0.5	0.5	0.7	0.4	0.4	0.4	0.3	1.3	0.9	0.9	1.7	1.4	1.0	0.9	3.1	2.6	2.2	1.1	1.2	1.5	1.8
Work status																									
Employed	3.4	3.0	1.8	1.3	1.3	0.9	1.2	1.0	0.7	0.5	0.4	4.4	2.8	1.9	4.5	4.0	2.8	2.4	6.8	7.1	6.2	2.8	3.3	3.2	3.4
Unemployed	2.5	2.6	2.3	2.4	2.4	0.8	1.2	1.7	1.6	1.1	0.9	1.8	2.8	1.7	4.3	4.3	3.5	4.0	na	7.0	5.8	4.2	5.1	5.0	4.4
Not in labour force	7.5	6.2	5.0	2.5	1.9	1.3	1.6	3.2	2.1	1.5	1.1	13.6	9.0	6.0	7.5	9.3	6.8	5.3	13.3	12.5	13.1	3.9	4.0	4.8	5.1
Country of birth																									
Born abroad	3.1	6.2	3.9	5.4	4.6	1.9	4.1	3.1	2.6	2.2	1.9	4.7	4.5	7.3	6.3	7.9	5.1	4.0	7.9	10.2	10.2	5.4	6.7	9.1	9.1
Native	5.9	4.8	3.3	1.6	1.3	0.4	0.9	2.1	1.4	0.9	0.6	9.6	6.7	4.2	6.2	7.1	5.0	4.0	9.6	10.1	10.4	3.2	3.3	3.2	3.1
Home tenure																									
Owned home	9.0	6.9	4.6	2.0	1.6	1.0	1.3	2.6	1.6	1.0	0.7	10.3	7.0	4.3	6.4	7.2	5.1	4.0	10.6	10.7	12.6	3.3	3.4	3.6	3.8
Not owned home	1.8	1.9	1.7	1.9	1.7	1.2	1.7	1.7	1.5	1.1	0.9	6.6	4.9	4.6	5.9	6.8	4.7	4.3	6.0	6.7	5.6	3.8	4.6	5.0	5.2
Total	5.8	4.9	3.4	1.9	1.6	1.1	1.4	2.2	1.5	1.0	0.8	9.6	6.7	4.4	6.2	7.1	5.0	4.0	9.6	10.1	10.4	3.4	3.7	3.9	4.1

All variables are dichotomous indicators (dummy variables)

### Sex and age

In Austria, England and Wales, France and Greece, the decline in the likelihood of coresidence in a three-generation household is apparent for each sex (although women have a higher likelihood than men), and is stronger at older ages (with the steepest declines in the oldest age groups in France and Greece) suggesting in these countries a change in the balance of dependency over time, from households where grandparents may require support from the younger generations to those where grandparents are more likely to be donors of support. By the same token, modest percentage increases in Romania and the US are mostly driven by rises in the 50–59 and 60–69 age groups.

### Marital status

We observe greater reductions in the percentage who are grandparents in a three-generation household among those separated, divorced, or widowed, suggesting that such households are increasingly meeting the needs of the younger rather than the older generation. This trend is seen broadly in all countries apart from Portugal, where the only group to show a consistent fall is the married. Similarly, in the US and Romania, the rise in three-generation households is mostly found among the married.

Even though three-generation households are becoming less common among the widowed and divorced/separated over time, widows and widowers were still more likely than those in other marital states to co-reside with their grandchild(ren). This holds in all countries at all time points studied, and suggests that the loss of one family relationship (or the end of one caring responsibility) tends to be compensated for by the strengthening of others.

### Education, employment status, and migration

In all countries studied, the trends are mainly driven by the lowest-attaining educational status group. In Romania, the percentage in three-generation households increased in the lowest educational group from 12% in 1977 to 17% in 2002 (but declined in the highest educational category). Similarly, in the US, the percentage among those with less than primary school education in such households rose from 8% in 1980 to 15% by 2010. In addition, in all the countries studied being a grandparent in a three-generation household is mostly associated with being outside the labour force (or unemployed in the US); and in all countries except Portugal and Romania, it is more likely in those born outside the country than among the native-born.

Turning from three-generation to skipped-generation households, Online Appendix 1 shows the characteristics of people aged 40 and over coresiding with their grandchild(ren) without a parent. As with three-generation households, the overall trends are consistently apparent for each of the characteristics considered, which have not varied greatly over time. A higher prevalence is observed among those with low education, not in paid work, and who do not own their dwelling, all suggestive of socio-economic disadvantage.

Table 2 shows grandparent household characteristics over time. With the exception of Austria, France, and Greece, around two-thirds of three-generation households were headed by a grandparent, suggesting that the younger generations had remained in, or moved to, a grandparental home with resources to share (Mutchler and Baker 2004). For most countries approaching half of three-generation households included both a grandmother and a grandfather, and this proportion increased over time. The age of the youngest grandparent was lower in England and Wales and the US than in the other countries. Whereas, the distribution in the number of grandchildren in three-generation households remained broadly similar over time, the age of the youngest grandchild became younger in England and Wales and France, though older in Portugal and Romania. These trends support the idea that such households are increasingly meeting the needs of the younger rather than the older generation.

**Table 2:** Household (HH) features, and grandparent (GP) and grandchild characteristics, grandparent households (either three- or skipped-generation), weighted percentages (Source: IPUMS-International (Minnesota Population Center 2017) and ONS LS)

Country	GP HH type	Year	Household headship		Household composition		Grandparent(s) in household			Age of youngest grandparent				Number of grandchildren			Age of youngest grandchild	
			GP	Parent	GPs and grandchildren only	Other(s) present	GM only	GF only	GM and GF	< 50	50–59	60–69	70+	1	2	3 or more	0–5	6–17
Austria	Three-generational	1981	40.6	59.4			48.9	10.2	40.9	10.9	22.2	26.0	40.8	48.7	29.0	22.3	49.9	50.1
		1991	50.2	49.8			42.0	9.5	48.4	13.9	25.2	30.3	30.6	55.4	29.2	15.4	55.5	44.5
		2001	39.8	60.2			40.2	11.5	48.4	12.1	23.0	30.4	34.6	53.1	34.0	13.0	51.1	48.9
	Skipped-generational	1981			84.9	15.2	44.0	4.2	51.8	6.5	32.8	37.6	23.1	86.7	11.0	2.3	13.5	86.6
		1991			80.5	19.5	43.3	6.1	50.6	8.0	30.5	34.8	26.8	87.9	10.1	2.0	26.2	73.8
		2001			87.6	12.4	35.7	6.9	57.4	7.1	32.9	38.4	21.6	87.1	11.6	1.3	13.5	86.5
England and Wales	Three-generational	1981	66.4	33.6			45.1	14.3	40.6	17.5	23.2	22.1	37.2	63.5	25.8	10.7	43.6	56.4
		1991	75.1	24.9			39.8	10.9	49.4	26.2	27.1	21.1	25.7	68.1	23.2	8.8	60.5	39.5
		2001	63.9	36.1			34.0	10.6	55.4	26.5	24.8	19.6	29.0	70.0	21.7	8.3	49.7	50.3
		2011	66.5	33.5			33.9	6.2	59.8	23.3	29.6	19.9	27.2	66.8	24.3	8.9	56.1	43.9
	Skipped-generational	1981			*84.6*	*15.5*	*35.2*	^a^	*61.8*	*7.1*	*37.3*	*43.1*	*12.4*	*86.7*	^a^	^a^	8.2	91.9
		1991			*86.7*	*13.3*	*36.3*	*4.6*	*59.2*	*13.7*	*36.6*	*39.7*	*10.1*	*87.5*	^a^	^a^	15.8	84.2
		2001			*86.7*	*13.3*	*33.1*	*4.6*	*62.2*	*13.4*	*41.6*	*34.2*	*10.7*	*82.0*	*13.3*	*4.6*	18.0	82.0
		2011			*81.3*	*18.7*	*40.4*	*4.7*	*54.9*	*11.6*	*37.9*	*36.1*	*14.5*	*78.6*	*16.7*	*4.7*	20.3	79.7
France	Three-generational	1982	22.6	77.4			54.6	12.9	32.5	8.0	19.6	24.2	48.2	53.4	30.1	16.6	37.5	62.5
		1990	29.7	70.3			50.1	11.6	38.3	11.7	22.7	29.4	36.2	59.7	27.2	13.2	46.0	54.0
		1999	30.1	69.9			49.6	11.1	39.3	12.9	23.7	28.6	34.8	61.2	27.4	11.4	51.2	48.9
		2011	32.9	67.1			49.2	10.9	39.9	10.9	27.5	29.6	32.0	60.4	28.1	11.5	56.7	43.3
	Skipped-generational	1982			87.5	12.5	30.3	2.1	67.6	6.0	38.7	37.7	17.7	84.8	12.1	3.2	12.6	87.4
		1990			87.7	12.3	26.7	1.9	71.4	8.5	30.9	46.7	13.9	85.0	12.4	2.6	14.6	85.4
		1999			87.9	12.1	32.7	2.9	64.4	6.7	28.1	46.1	19.1	83.7	13.7	2.7	11.7	88.3
		2011			76.5	23.5	32.8	5.2	62.1	3.7	8.4	35.1	52.8	88.0	10.4	1.6	19.6	80.4
Greece	Three-generational	1981	29.0	71.0			52.3	10.4	37.3	4.5	16.4	30.8	48.4	34.9	46.7	18.5	48.9	51.1
		1991	33.5	66.5			49.5	10.6	39.9	3.7	17.9	32.1	46.3	39.6	45.2	15.3	*41.0*	*59.0*
		2001	39.4	60.6			48.2	9.7	42.2	5.2	17.3	36.8	40.7	47.4	40.4	12.2	43.3	56.7
	Skipped-generational	1981			86.3	13.7	27.9	2.4	69.7	9.3	36.6	36.7	17.4	73.4	23.0	3.6	38.1	61.9
		1991			84.8	15.2	25.6	4.4	70.1	8.0	38.2	36.9	16.9	72.0	22.5	5.5	*38.6*	*61.4*
		2001			65.6	34.4	29.5	5.8	64.7	7.1	27.4	43.3	22.2	72.9	21.5	5.6	38.5	61.5
Portugal	Three-generational	1981	67.5	32.5			38.6	10.0	51.4	14.7	29.5	27.6	28.2	55.6	29.6	14.9	63.2	36.8
		1991	55.5	44.5			47.3	10.7	41.9	9.1	23.8	32.4	34.7	56.7	32.0	11.3	46.7	53.3
		2001	63.1	36.9			46.4	9.9	43.7	10.6	21.7	31.9	35.8	66.2	27.1	6.6	47.5	52.5
		2011	70.3	29.7			43.9	8.4	47.7	11.3	26.2	28.0	34.6	69.4	25.3	5.3	46.1	53.9
	Skipped-generational	1981			75.5	24.5	35.8	2.9	61.3	5.4	32.0	41.7	20.9	75.7	19.3	5.0	27.7	72.3
		1991			79.1	20.9	31.4	3.8	64.8	5.8	28.4	44.8	21.0	80.4	16.1	3.6	23.0	77.0
		2001			75.9	24.1	35.4	2.1	62.5	6.1	29.4	41.3	23.2	81.1	13.9	5.0	21.5	78.5
		2011			72.3	27.7	32.1	2.6	65.3	5.0	29.4	40.2	25.4	81.3	16.2	2.5	21.3	78.7
Romania	Three-generational	1977	45.0	55.0			46.5	8.8	44.8	15.0	25.0	33.3	26.7	50.0	34.0	16.0	59.5	40.5
		1992	74.5	25.5			39.6	8.1	52.3	15.9	32.2	30.2	21.7	53.5	31.2	15.4	59.1	40.9
		2002	80.7	19.3			41.2	8.9	49.9	13.9	30.6	34.8	20.8	61.1	30.3	8.6	50.1	49.9
	Skipped-generational	1977			86.3	13.7	22.8	3.0	74.2	21.8	38.4	31.4	8.4	86.1	11.8	2.1	46.0	54.0
		1992			87.4	12.6	26.9	2.9	70.2	9.7	41.0	38.8	10.5	82.9	14.3	2.9	29.6	70.4
		2002			87.4	12.6	32.5	3.5	64.0	10.1	37.2	38.9	13.8	82.9	14.6	2.5	20.8	79.2
USA	Three-generational	1980	61.8	38.2			51.9	10.2	37.9	24.4	28.9	24.3	22.3	56.0	28.2	15.9	54.8	45.2
		1990	72.0	28.0			49.2	8.6	42.3	27.8	29.9	25.3	17.0	57.2	27.8	15.0	59.1	40.9
		2000	67.8	32.2			49.1	10.1	40.8	27.6	29.2	23.5	19.8	56.5	28.6	14.9	57.1	42.9
		2010	67.3	32.7			47.3	10.0	42.7	23.2	32.9	26.1	17.9	54.8	29.7	15.6	58.3	41.7
	Skipped-generational	1980			77.1	22.9	37.8	4.1	58.1	13.7	37.6	34.5	14.3	75.3	17.3	7.4	20.4	79.6
		1990			75.0	25.0	41.7	4.0	54.3	18.4	36.8	30.7	14.2	74.4	18.1	7.5	26.4	73.6
		2000			76.9	23.1	41.5	4.8	53.7	19.3	39.6	27.8	13.2	72.5	19.0	8.6	24.3	75.8
		2010			73.3	26.7	42.4	5.9	51.7	17.0	37.8	31.2	14.0	70.1	21.0	8.9	28.4	71.7

Italicised percentages are *not significant*. All other percentages are significant at *p* < 0.01

^a^Percentage withheld by Office for National Statistics because small cell counts offer risk of disclosure

In contrast to three-generation households, skipped-generation households were even more likely to include both grandparents for the most recent time point. In England and Wales, France and the US, these households have become more likely to include at least one grandchild aged 0–5, although the reverse was the case for Portugal and Romania.

### Multivariate analysis

Table 3 shows results for the multinomial regression model for three time points up to around 2001. Most countries, unlike the US, exhibited a decline in the likelihood of grandparenthood in either type of household even when socio-economic and demographic factors were considered. However, three-generation grandparenthood showed a significant increase in Romania with the risk in 2002 being 26% higher than in 1977; and England and Wales showed a significant rise in skipped-generation grandparenthood, though the prevalence is very low.

**Table 3:** Multinomial logit regressions of being aged 40 or over and living with a grandchild in (1) a three-generation or (2) a skipped-generation household: Austria, England and Wales, France, Greece, Portugal, Romania and US up to ~2001 only (Source: IPUMS-International (Minnesota Population Center 2017) and ONS LS)

	HH type	Austria	England and Wales	France	Greece	Portugal	Romania	US
		Odds ratio	Odds ratio	Odds ratio	Odds ratio	Odds ratio	Odds ratio	Odds ratio
Sex (female)^a^	Three	0.89	0.93	1.10	1.12	1.07	1.08	1.36
	Skipped	1.12	*1.14*	1.12	1.12	1.19	1.19	1.39
Age	Three	1.28	1.21	1.26	1.51	1.37	1.43	1.29
	Skipped	1.77	2.25	2.11	1.94	2.03	2.08	1.72
Age squared	Three	1.00	1.00	1.00	1.00	1.00	1.00	1.00
	Skipped	1.00	0.99	0.99	0.99	0.99	0.99	1.00
Marital status^b^								
Never married	Three	0.32	0.44	0.51	0.05	0.37	0.45	0.58
	Skipped	0.31	0.16	0.19	0.05	0.27	0.34	0.46
Divorced/sep.	Three	*0.98*	1.49	1.30	*1.00*	1.23	0.89	1.41
	Skipped	0.82	*0.95*	0.54	0.61	0.70	0.53	0.91
Widowed	Three	1.66	3.18	2.59	2.06	2.37	1.74	1.96
	Skipped	0.88	*0.86*	0.75	0.82	*0.98*	0.71	0.93
Educational^c^								
Less than primary education	Three	*Na*	*na*	4.80	5.88	5.04	3.21	5.95
	Skipped	*Na*	*na*	2.77	3.96	4.00	1.89	6.81
Primary	Three	6.01	2.92	2.52	3.91	2.60	2.62	3.59
	Skipped	2.18	2.27	1.83	2.63	2.05	1.67	4.49
Secondary	Three	2.48	1.50	1.65	1.43	1.63	1.56	2.25
	Skipped	1.76	1.85	1.27	*1.17*	1.50	1.35	2.52
Employment^d^								
Unemployed	Three	1.15	1.38	1.63	*1.09*	1.19	1.27	1.22
	Skipped	*1.25*	*1.34*	1.67	*0.94*	*1.01*	*0.87*	1.21
Not active	Three	1.07	1.24	1.38	1.18	1.16	1.24	1.14
	Skipped	1.33	1.75	1.56	1.14	1.17	1.34	1.12
Born abroad^e^	Three	1.82	4.19	1.97	2.20	1.81	*0.98*	2.42
	Skipped	1.22	*1.03*	0.83	*1.30*	1.75	*0.98*	0.59
Not owned dwelling^f^	Three	0.22	0.87	0.71	0.85	*0.98*	0.63	*1.00*
	Skipped	1.60	2.15	1.55	0.80	1.54	0.90	1.38
Census year^g^								
1990s	Three	0.86	0.87	*1.00*	0.64	1.35	1.07	0.97
	Skipped	0.79	*1.15*	1.36	0.58	1.12	1.26	*0.98*
2000s	Three	0.63	0.36	0.79	0.40	*1.01*	1.26	1.14
	Skipped	0.46	1.64	0.92	0.50	0.81	0.73	1.40

Italicised coefficients are not significant. All other coefficients at *p* < 0.01

Reference categories are: (1) male^a^; (2) married or cohabiting^b^; (3) university education^c^; (4) employed^d^; (5) native born^e^; (6) owned dwelling^f^; and (7) 1980s^g^

In the European countries studied (as in the US), living with a grandchild appeared to be associated with socio-economic disadvantage as represented by women, those previously married, those with lower educational levels, and the unemployed or retired. In most countries those born abroad, an increasing proportion of the population from the 1980s onward, were more likely than the native-born to form three-generation households (Castles and Miller 2002); however, in the US being a grandparent in a skipped-generation household was more common among the native-born.

Online Appendix 2 shows the results from a similar analysis conducted for those countries with more recent data (that is for England and Wales, France, Portugal and the US to 2010/2011). Overall, the findings are similar to those shown in Table 3, though in England and Wales the declining percentage in three-generation households began to rise again between 2001 and 2011, potentially reflecting economic conditions.

## Discussion

We studied six European countries as well as the US: Austria, England and Wales, France, Greece, Portugal, and Romania. Given variations in policy environments, we had expected grandparent households to increase in Greece and Portugal—familistic societies—and in Romania as it is characterised by supported familialism. However, most countries showed a significant decline in the percentage of people aged 40 and over residing with a grandchild in a three-generation household since the late 1970s/early 1980s. Only Romania showed an increase like the US. These trends remained significant even when demographic and socio-economic factors were considered.

Thus, our research has tracked trends in intergenerational coresidence showing changing patterns across time and in different societies. Our findings show that in all the countries studied grandparental coresidence (as the US literature suggests) are generally associated with socio-economic disadvantage, being more prevalent among women, the widowed, those with lower education, those not working, and those born abroad. In some cases, this association became stronger over time. For example, in Romania and the US rises in three-generation households were primarily driven by grandparents with the lowest educational levels suggesting that such households may be increasingly used as an adaptive strategy among families with the fewest resources.

Regardless of overall trends, grandparent households in the countries studied appear to be becoming more common among grandparents able to provide rather than in need of support, since they are becoming younger (or were already younger, in Romania and the US) and more likely to be married. The fall in the age of the youngest grandchild in three-generation households, noticeable in England and Wales, France and the US, may reinforce this impression of supportive rather than supported grandparenthood for those countries.

In the US, as noted above, rises in skipped-generation households have been attributed to increasing parental inability to care for children due, for example, to drug or alcohol misuse (Baker et al. 2008; Copen 2006; Cuddeback 2004; Goodman et al. 2004; Goodman and Silverstein 2001, 2002; Hayslip and Kaminski 2005; Jendrek 1993; Minkler 1999; Minkler and Roe 1996; Pebley and Rudkin 1999; Smith and Beltran 2001), and the rise in maternal (and paternal) imprisonment (Goodman and Silverstein 2002; Turney 2014). In addition, evidence suggests that the opioid epidemic in the US may be responsible for recent increases in the number of children in the care of relatives, many of whom are placed with grandparents (Generations United 2016). In a rare study in Europe, the rise in the low prevalence of skipped-generation households in the UK since the 1980s is also attributed to increases in parental drug and alcohol misuse and imprisonment (Nandy et al. 2011).

Financial hardship is seen as another important reason for drawing on the support of extended family in the form of intergenerational coresidence (Baker et al. 2008; Copen 2006; Goodman and Silverstein 2002; Minkler 1999). For example, by comparison with single-mother households, those with a co-resident grandparent are usually better off financially (Goodman and Silverstein 2002; Jendrek 1993; Mutchler and Baker 2009; Tienda and Angel 1982). Moreover, in the US increases in multigenerational households have been attributed, in part, to the financial crisis brought on by the Great Recession in 2007–2009 (Swartz 2009; Wiemers 2014).

The widespread economic austerity in Europe in the late 1980s and early 1990s (and more recently starting between 2008 and 2010) would lead us to also expect an increase in grandparent households for the same reasons as in the US—especially in familistic countries like Greece and Portugal with few alternatives to family support (Saraceno and Keck 2010). However, while three-generation households are more common in the poorer European countries (Fig. 1), only in Romania was there a significant increase in such households. The findings for Greece and Portugal require further investigation with more recent census data as the economic crisis lasted until about 2016 in the former country and until around 2014 in the latter. Although Romania is characterised by supported familialism, social assistance benefits to families are largely tied to earnings thereby favouring middle class working parents with only modest benefits available to low-income jobless families or those with irregular work histories, leading to greater reliance on relatives (Inglot et al. 2012; Preoteasa et al. 2018). Together with the collapse of many state provided services in former socialist countries, rising poverty and child poverty in particular in the late 1980s and 1990s, is also likely to have led to a greater reliance on families (Bezemer 2006). In Romania, just over 50% of children are at risk of poverty or social exclusion, one of the highest levels in Europe, compared to 12–19% for the Nordic countries (Save the Children 2014). Thus, the rise in grandparent households in this country may reflect a coping strategy among poorer families to increasing financial hardship (Preoteasa et al. 2018; Stephens et al. 2015). In addition, rising poverty has meant that many Romanians have migrated abroad for work often leaving the extended family to look after the children left behind (Inglot et al. 2012; Piperno 2012).

Policy changes are also believed to play a role, particularly in the US (Baker et al. 2008; Smith and Beltran 2001). Especially significant have been reforms ending entitlement to welfare benefits except under strict restrictions (such as work requirements), or for teenage mothers, making receipt of benefits conditional on residence with a parent and enrolment in education—a strong incentive for multigenerational living (Baker et al. 2008; Smith and Beltran 2001). It may not be surprising that grandparental coresidence is highest in the US given the relatively high rates of teenage births in comparison to those in Europe despite recent declines (e.g. the birth rate is 34 per 1000 females aged 15–19 in the US and only 7 per 1000 in France) (Sedgh et al. 2015). Moreover, the US also experienced significant changes to its child welfare system: a shift toward more children being placed in formal kinship care (usually with grandparents) than in foster care (Baker et al. 2008; Smith and Beltran 2001). Such changes are thought to have contributed to enhancing the role of grandparents in kinship care in the US.

Similar policy shifts toward family rather than foster care have also occurred in the UK. For example, formal kinship care is believed to have increased since the introduction of the 1989 Children Act which mandated that children should be placed with kin in preference to other placements, a trend reinforced by subsequent legislation (Nandy and Selwyn 2013). These policy changes are thought to be responsible in part for the increase in the low prevalence of skipped-generation households witnessed in the UK.

Little work outside the US has attempted to explain trends in grandparent households in relation to the two theories discussed above—that is a latent matrix of kin activation and structural lag (Baker et al. 2008). The continuing increases in in three- and skipped-generation households in the US support the activation of a latent network of kin in times of family crisis and in response to the retrenchment of state provided services (Cherlin and Seltzer 2014)—a response which may act to perpetuate inequalities (Saraceno 2016; Swartz 2009; Tienda and Angel 1982). The insufficient recognition of intergenerational ties in family policies in both Europe and the US represents structural lag: the asynchrony between changing family forms and social policies.

Due to the limitations in the data discussed earlier, we were only able to examine co-resident grandparenthood in a restricted number of European countries, at intervals of around a decade (and for some countries only three rather than four time points were available at the time the study was conducted). In all census data, the covariates available for investigation are few; most notable, of course, is the lack of information on health status and on any support given or received. Nevertheless, the advantages to using census microdata lie in the larger sample numbers and the greatly improved coverage of the population.

Finally, delays in the timing of grandparenthood in many European countries may be contributing to an apparent decline in grandparental coresidence (Leopold and Skopek 2015). Despite these limitations, to our knowledge this is the first study to examine patterns in adults living with a grandchild from a cross-national comparative perspective over time. Future research would benefit from using the most recent census data for all the countries studied, if and when available, to see if patterns of intergenerational coresidence noted here continue.

## Electronic supplementary material

Below is the link to the electronic supplementary material.

Supplementary material 1 (DOCX 47 KB)
