# Supplementary material for: Trends in the prevalence of grandparents living with grandchild(ren) in selected European countries and the United States
Source: Eur J Ageing. 2018 May 23;15(3):237–50. doi: 10.1007/s10433-018-0474-3 (PMC6156723; doi:10.1007/s10433-018-0474-3)
Supplement: Supplementary file 1 — Supplementary material 1 (DOCX 47 KB) [file 10433_2018_474_MOESM1_ESM.docx]

**Appendix 1: Percentage of people aged 40 and over living with grandchild(ren) aged 0-17 but no child, by selected adult characteristics, weighted data.**

|  |  | Austria | | | England and Wales | | | | France | | | | Greece | | | Portugal | | | | Romania | | | USA | | | |
| --- | --- | --- | --- | --- | --- | --- | --- | --- | --- | --- | --- | --- | --- | --- | --- | --- | --- | --- | --- | --- | --- | --- | --- | --- | --- | --- |
|  |  | 1981 | 1991 | 2001 | 1981 | 1991 | 2001 | 2011 | 1982 | 1990 | 1999 | 2011 | 1981 | 1991 | 2001 | 1981 | 1991 | 2001 | 2011 | 1977 | 1992 | 2002 | 1980 | 1990 | 2000 | 2010 |
|  |  | % | % | % | % | % | % | % | % | % | % | % | % | % | % | % | % | % | % | % | % | % | % | % | % |  |
| SEX | Male | 0.4 | 0.3 | 0.2 | 0.1 | 0.1 | 0.2 | 0.2 | 0.2 | 0.2 | 0.1 | 0.0 | 0.5 | 0.3 | 0.3 | 0.9 | 0.7 | 0.5 | 0.3 | 0.8 | 1.1 | 0.5 | 0.5 | 0.5 | 0.6 | 0.6 |
|  | Female | 0.6 | 0.4 | 0.3 | 0.2 | 0.2 | 0.2 | 0.2 | 0.3 | 0.2 | 0.1 | 0.0 | 0.6 | 0.4 | 0.3 | 1.1 | 0.8 | 0.6 | 0.4 | 0.9 | 1.2 | 0.6 | 0.6 | 0.7 | 0.9 | 0.9 |
| AGE | 40s | 0.1 | 0.1 | 0.0 | 0.0 | 0.1 | 0.1 | 0.1 | 0.0 | 0.0 | 0.0 | 0.0 | 0.1 | 0.1 | 0.1 | 0.1 | 0.1 | 0.1 | 0.1 | 0.4 | 0.3 | 0.1 | 0.2 | 0.3 | 0.3 | 0.3 |
|  | 50s | 0.6 | 0.5 | 0.3 | 0.2 | 0.3 | 0.3 | 0.3 | 0.3 | 0.2 | 0.1 | 0.0 | 0.6 | 0.4 | 0.3 | 1.0 | 0.7 | 0.6 | 0.4 | 1.3 | 1.5 | 0.8 | 0.7 | 1.0 | 1.2 | 0.9 |
|  | 60s | 0.9 | 0.6 | 0.4 | 0.3 | 0.3 | 0.4 | 0.3 | 0.5 | 0.3 | 0.2 | 0.0 | 1.1 | 0.6 | 0.5 | 2.0 | 1.6 | 1.0 | 0.7 | 1.4 | 2.1 | 1.1 | 0.9 | 1.0 | 1.3 | 1.2 |
|  | 70s | 0.6 | 0.5 | 0.3 | 0.2 | 0.1 | 0.2 | 0.2 | 0.3 | 0.2 | 0.1 | 0.1 | 0.7 | 0.4 | 0.4 | 1.5 | 1.0 | 0.7 | 0.5 | 0.7 | 1.1 | 0.5 | 0.5 | 0.5 | 0.7 | 0.8 |
|  | 80s | 0.2 | 0.4 | 0.1 | 0.0 | 0.0 | 0.0 | 0.1 | 0.1 | 0.1 | 0.0 | 0.0 | 0.3 | 0.2 | 0.2 | 0.6 | 0.5 | 0.3 | 0.3 | 0.2 | 0.3 | 0.2 | 0.2 | 0.3 | 0.3 | 0.3 |
|  | 90 plus | 0.2 | 0.4 | 0.1 | 0.0 | 0.0 | 0.0 | 0.0 | 0.0 | 0.0 | 0.0 | 0.0 | 0.1 | 0.0 | 0.0 | 0.3 | 0.1 | 0.2 | 0.3 | 0.2 | 0.1 | 0.1 | 0.1 | 0.1 | 0.2 | 0.1 |
| MARITAL STATUS | Never-married | 0.1 | 0.2 | 0.1 | 0.0 | 0.0 | 0.0 | 0.1 | 0.0 | 0.1 | 0.0 | 0.0 | 0.0 | 0.0 | 0.0 | 0.3 | 0.2 | 0.1 | 0.1 | 0.2 | 0.4 | 0.2 | 0.1 | 0.3 | 0.4 | 0.4 |
|  | Married | 0.5 | 0.4 | 0.3 | 0.2 | 0.2 | 0.2 | 0.2 | 0.3 | 0.2 | 0.1 | 0.0 | 0.6 | 0.4 | 0.3 | 1.0 | 0.8 | 0.5 | 0.4 | 1.0 | 1.3 | 0.6 | 0.5 | 0.7 | 0.8 | 0.8 |
|  | Divorced/Sep | 0.4 | 0.3 | 0.2 | 0.2 | 0.2 | 0.2 | 0.3 | 0.2 | 0.1 | 0.1 | 0.0 | 0.3 | 0.2 | 0.1 | 1.0 | 0.6 | 0.4 | 0.3 | 0.5 | 0.5 | 0.4 | 0.5 | 0.7 | 0.8 | 0.8 |
|  | Widowed | 0.6 | 0.5 | 0.3 | 0.2 | 0.2 | 0.1 | 0.2 | 0.2 | 0.2 | 0.1 | 0.0 | 0.6 | 0.3 | 0.3 | 1.3 | 0.9 | 0.7 | 0.4 | 0.7 | 1.0 | 0.6 | 0.6 | 0.8 | 0.8 | 0.8 |
| EDUCATION | Less than primary | na | na | na | na | na | na | na | 0.3 | 0.2 | 0.1 | 0.0 | 0.8 | 0.6 | 0.5 | 1.1 | 0.8 | 0.6 | 0.5 | 1.0 | 1.5 | 0.8 | 1.4 | 1.6 | 1.3 | 1.1 |
|  | Primary | 0.6 | 0.5 | 0.3 | 0.2 | 0.2 | 0.2 | 0.2 | 0.2 | 0.2 | 0.1 | 0.0 | 0.5 | 0.3 | 0.3 | 0.5 | 0.4 | 0.3 | 0.2 | 0.8 | 1.1 | 0.7 | 0.7 | 1.2 | 1.5 | 1.4 |
|  | Secondary | 0.4 | 0.3 | 0.2 | 0.1 | 0.1 | 0.2 | 0.2 | 0.1 | 0.1 | 0.1 | 0.0 | 0.2 | 0.1 | 0.1 | 0.5 | 0.4 | 0.2 | 0.1 | 0.7 | 0.7 | 0.4 | 0.3 | 0.5 | 0.8 | 0.8 |
|  | University | 0.2 | 0.2 | 0.1 | 0.0 | 0.1 | 0.1 | 0.1 | 0.1 | 0.1 | 0.1 | 0.0 | 0.2 | 0.1 | 0.1 | 0.4 | 0.3 | 0.1 | 0.1 | 0.5 | 0.6 | 0.3 | 0.2 | 0.2 | 0.3 | 0.3 |
| WORK STATUS | Employed | 0.3 | 0.2 | 0.1 | 0.1 | 0.1 | 0.1 | 0.1 | 0.1 | 0.1 | 0.0 | 0.0 | 0.4 | 0.2 | 0.1 | 0.6 | 0.4 | 0.3 | 0.2 | 0.8 | 0.8 | 0.3 | 0.4 | 0.5 | 0.6 | 0.6 |
|  | Unemployed | 0.2 | 0.3 | 0.2 | 0.2 | 0.1 | 0.2 | 0.2 | 0.3 | 0.2 | 0.1 | 0.0 | 0.1 | 0.2 | 0.2 | 0.5 | 0.6 | 0.4 | 0.3 | na | 0.5 | 0.2 | 0.5 | 0.7 | 0.8 | 0.7 |
|  | Not in labour force | 0.7 | 0.5 | 0.3 | 0.2 | 0.2 | 0.3 | 0.3 | 0.3 | 0.2 | 0.2 | 0.0 | 0.6 | 0.4 | 0.4 | 1.3 | 1.0 | 0.7 | 0.5 | 1.0 | 1.5 | 0.8 | 0.6 | 0.8 | 0.9 | 0.9 |
| COUNTRY OF BIRTH | Born abroad | 0.3 | 0.5 | 0.3 | 0.2 | 0.2 | 0.2 | 0.2 | 0.2 | 0.2 | 0.1 | 0.0 | 0.3 | 0.2 | 0.2 | 1.2 | 0.7 | 0.6 | 0.4 | 0.9 | 1.3 | 0.6 | 0.3 | 0.4 | 0.5 | 0.5 |
|  | Native | 0.5 | 0.4 | 0.2 | 0.2 | 0.2 | 0.2 | 0.2 | 0.3 | 0.2 | 0.1 | 0.0 | 0.5 | 0.4 | 0.3 | 1.0 | 0.8 | 0.5 | 0.4 | 0.9 | 1.2 | 0.6 | 0.5 | 0.7 | 0.8 | 0.8 |
| HOME TENURE | Owned home | 0.3 | 0.3 | 0.2 | 0.1 | 0.1 | 0.2 | 0.1 | 0.2 | 0.2 | 0.1 | 0.0 | 0.6 | 0.4 | 0.3 | 0.9 | 0.7 | 0.5 | 0.3 | 1.0 | 1.2 | 0.6 | 0.5 | 0.6 | 0.7 | 0.7 |
|  | Not owned home | 0.7 | 0.5 | 0.3 | 0.3 | 0.3 | 0.3 | 0.3 | 0.3 | 0.2 | 0.1 | 0.0 | 0.3 | 0.3 | 0.2 | 1.1 | 0.9 | 0.7 | 0.6 | 0.6 | 0.9 | 0.5 | 0.6 | 0.8 | 1.0 | 0.9 |
|  | Total | 0.5 | 0.4 | 0.2 | 0.2 | 0.2 | 0.2 | 0.2 | 0.2 | 0.2 | 0.1 | 0.0 | 0.5 | 0.3 | 0.3 | 1.0 | 0.8 | 0.5 | 0.4 | 0.9 | 1.2 | 0.6 | 0.5 | 0.7 | 0.8 | 0.7 |

Source: IPUMS-International (Minnesota Population Center, 2017) and ONS Longitudinal Study.

*Notes*: All variables are dichotomous indicators (dummy variables).

**Appendix 2: Multinomial logit regressions of being aged 40 or over and living with a grandchild in (1) a three-generation or (2) a skipped-generation household: England and Wales, France, Portugal, and US including 2010/11**

|  |  | England & Wales | France | Portugal | US |
| --- | --- | --- | --- | --- | --- |
|  | House-hold Type | Odds ratio | Odds ratio | Odds ratio | Odds ratio |
| **Sex (female)^a^** | Three | *0.02* | 1.11 | 1.08 | 1.36 |
|  | Skipped | 1.21 | 1.16 | 1.20 | 1.41 |
| **Age** | Three | 1.26 | 1.28 | 1.37 | 1.29 |
|  | Skipped | 2.15 | 2.15 | 2.03 | 1.73 |
| **Age squared** | Three | 1.00 | 1.00 | 1.00 | 1.00 |
|  | Skipped | 0.99 | 0.99 | 0.99 | 1.00 |
| **Marital status^b^** |  |  |  |  |  |
| Never married | Three | 0.44 | 0.54 | 0.37 | 0.54 |
|  | Skipped | 0.22 | 0.22 | 0.26 | 0.42 |
| Divorced/  separated | Three | 1.24 | 1.43 | 1.30 | 1.34 |
|  | Skipped | *0.93* | 0.73 | *0.83* | 0.83 |
| Widowed | Three | 2.70 | 2.51 | 2.35 | 1.99 |
|  | Skipped | *0.85* | 0.71 | *0.95* | 0.95 |
| **Educational^c^** |  |  |  |  |  |
| Less than primary education | Three | *na* | 3.79 | 4.70 | 6.72 |
|  | Skipped | *na* | 1.45 | 3.24 | 8.84 |
| Primary | Three | 2.61 | 2.16 | 2.58 | 4.08 |
|  | Skipped | 2.07 | *1.14* | 1.97 | 5.70 |
| Secondary | Three | 1.67 | 1.59 | 1.60 | 2.31 |
|  | Skipped | 1.64 | *1.12* | *1.39* | 2.65 |
| **Employment^d^** |  |  |  |  |  |
| Unemployed | Three | 1.26 | 1.73 | 1.26 | 1.16 |
|  | Skipped | *1.33* | 1.88 | *1.23* | 1.11 |
| Not active | Three | 1.20 | 1.37 | 1.16 | 1.14 |
|  | Skipped | 1.73 | 1.57 | 1.18 | 1.12 |
| **Foreign-born^e^** |  |  |  |  |  |
| Born abroad | Three | 4.34 | 2.09 | 1.85 | 2.27 |
|  | Skipped | *1.01* | *0.97* | 1.84 | 0.51 |
| **Home ownership^f^** |  |  |  |  |  |
| Not own dwelling | Three | 0.95 | 0.70 | 0.96 | *0.99* |
|  | Skipped | 2.24 | 1.42 | 1.42 | 1.38 |
| **Census year^g^** |  |  |  |  |  |
| 1990s | Three | 0.88 | 0.75 | 1.16 | 1.33 |
|  | Skipped | *1.15* | 0.70 | 0.77 | 1.69 |
| 2000s | Three | 0.35 | 0.58 | 0.86 | 1.59 |
|  | Skipped | 1.67 | 0.43 | 0.56 | 2.51 |
| 2010s | Three | 0.83 | 0.50 | 0.74 | 1.69 |
|  | Skipped | 1.71 | 0.79 | 0.42 | 2.52 |

Source: IPUMS-International (Minnesota Population Center, 2017) and ONS Longitudinal Study.

Note: Italicised coefficients are not significant. All other coefficients at p<.01.

Reference categories are: (a) male; (b) married or cohabiting; (c) university education; (d) employed; (e) native born; (f) own dwelling; and (g) 1980s.
